# Automatic evaluation of tumor budding in immunohistochemically stained colorectal carcinomas and correlation to clinical outcome

**DOI:** 10.1186/s13000-018-0739-3

**Published:** 2018-08-28

**Authors:** Cleo-Aron Weis, Jakob Nikolas Kather, Susanne Melchers, Hanaa Al-ahmdi, Marion J. Pollheimer, Cord Langner, Timo Gaiser

**Affiliations:** 1 Institute of Pathology, University Medical Centre Mannheim, University of Heidelberg, 68167 Mannheim, Germany; 2 Department of Medical Oncology and Internal Medicine VI, National Center for Tumor Diseases, University Hospital Heidelberg, Heidelberg University, Heidelberg, Germany; 3 Department of Dermatology, Venereology and Allergology, University Medical Center Mannheim, Heidelberg University, Mannheim, Germany; 4 Institute of Pathology, Medical University Graz, Graz, Austria

**Keywords:** Colorectal carcinoma, Digital pathology, Tumor budding, Image processing, Convolutional neural network

## Abstract

**Background:**

Tumor budding, meaning a detachment of tumor cells at the invasion front of colorectal carcinoma (CRC) into single cells or clusters (<=5 tumor cells), has been shown to correlate to an inferior clinical outcome by several independent studies. Therefore, it has been discussed as a complementary prognostic factor to the TNM staging system, and it is already included in national guidelines as an additional prognostic parameter. However, its application by manual evaluation in routine pathology is hampered due to the use of several slightly different assessment systems, a time-consuming manual counting process and a high inter-observer variability. Hence, we established and validated an automatic image processing approach to reliably quantify tumor budding in immunohistochemically (IHC) stained sections of CRC samples.

**Methods:**

This approach combines classical segmentation methods (like morphological operations) and machine learning techniques (k-means and hierarchical clustering, convolutional neural networks) to reliably detect tumor buds in colorectal carcinoma samples immunohistochemically stained for pan-cytokeratin. As a possible application, we tested it on whole-slide images as well as on tissue microarrays (TMA) from a clinically well-annotated CRC cohort.

**Results:**

Our automatic tumor budding evaluation tool detected the absolute number of tumor buds per image with a very good correlation to the manually segmented ground truth (R2 value of 0.86).

Furthermore the automatic evaluation of whole-slide images from 20 CRC-patients, we found that neither the detected number of tumor buds at the invasion front nor the number in hotspots was associated with the nodal status. However, the number of spatial clusters of tumor buds (budding hotspots) significantly correlated to the nodal status (*p*-value = 0.003 for N0 vs. N1/N2). TMAs were not feasible for tumor budding evaluation, as the spatial relationship of tumor buds (especially hotspots) was not preserved.

**Conclusions:**

Automatic image processing is a feasible and valid assessment tool for tumor budding in CRC on whole-slide images. Interestingly, only the spatial clustering of the tumor buds in hotspots (and especially the number of hotspots) and not the absolute number of tumor buds showed a clinically relevant correlation with patient outcome in our data.

**Electronic supplementary material:**

The online version of this article (10.1186/s13000-018-0739-3) contains supplementary material, which is available to authorized users.

## Declaration of presentation of findings at a conference

This study was presented in part at the 101st annual meeting of the German Society of Pathology (DGP) in Berlin, Germany in May 2018 and will be presented at the 30th European Congress of Pathology in Bilbao, Spain, in September 2018.

## Background

### Tumor budding as an additional prognostic parameter in colorectal cancer

The most commonly applied clinicopathological staging system for colorectal cancer (CRC), which is one of the most frequent solid tumors worldwide [1], is the T (tumor) N (lymph nodes) M (metastases) staging system, which classifies tumors based on primary tumor extension, regional nodal involvement and the absence or presence of metastases [2, 3].

Despite this complex and multivariate staging system, there is still room for improvement. On one hand, cases with a low T or N stage sometimes show distant metastasis, while on the other hand, high T or N stage tumors can exhibit an uneventful clinical course [4–6]. For cases classified as intermediate according to TNM, prognostic statements are nearly impossible. It is therefore generally agreed that new features or molecular markers allowing a better stratification are necessary [6].

One morphological feature that is discussed to close this gap is the concept of tumor budding, which goes back to the 1950s, when Imai postulated the existence and biological relevance of detachment of tumor cells at the invasion front [7]. For this feature—despite the different concepts around how to define tumor budding, the problem of how to evaluate budding and the huge inter-observer variance—many studies in general, and in particular for CRC [8], have been able to show a correlation to clinical outcome and to the likelihood of nodal positivity [9, 10].

### Different tumor budding definitions and assessment approaches

The morphological feature “tumor budding” was first described in the 1950s and showed a correlation to clinical outcome in many studies with different analytical approaches (e.g., visual assessment vs. image processing) [4, 5, 7, 9–13], especially in colorectal carcinoma. This is notable since the definition of tumor buds is not trivial and there have been many discussions about how to assess budding.

Most research projects on tumor budding in CRC defined a tumor bud as a cluster of a few (in most studies less than 5 neighboring cells) poorly or dedifferentiated tumor cells in the desmoplastic stroma that are detached from larger tumor islands. This more or less arbitrary definition goes back to works from Gabbert et al. [14] and Hase et al. [8]. They defined tumor buds as clusters of tumor cells with a distinct morphology that could be described as epithelial-mesenchymal transition or focal dedifferentiation [14, 15]. Obviously, this has led to confusion with dedifferentiated morphology in the sense of the WHO grading and is also difficult to discriminate from a diffuse infiltration pattern [1, 8, 16].

Concerning the assessment of tumor budding, there are different approaches throughout the literature. Most approaches include focusing on hotspots, without explicitly defining them, and subsequent evaluation of the numbers of tumor buds. For instance, the German S3 guidelines from 2017, which included tumor budding as an additional risk factor for nodal positivity in early colorectal cancer, gives the following recommendations [17–21]: 1) the invasion front should be scanned at low magnification for the area of the highest tumor budding (“hottest spot”) [17, 21]; 2) in this area, the absolute number of tumor buds should be counted [17, 21]; 3) the tumor should be graded based on the number of buds (grade 1 with 0–4 buds, grade 2 with 5–9 buds and grade 3 with < 9 buds) [17, 21].

### Study aims

Although tumor budding evaluation is a painstaking counting task, there are only a few works focusing on automatization. In the context of CRC, there is only one work from Caie et al. on automatic tumor budding quantification [12]. The vast majority of works rely on human evaluation with the abovementioned problems of low inter-observer correlation. Additionally, this is very time consuming and requires intensive training.

Against this background, we here establish an automatic image processing approach to reliably quantify tumor budding in immunohistochemically (IHC) stained sections of CRC samples. By publicly sharing all source codes, we hope to enable others to reproduce our results and apply it in their own scientific work or on routine histology sections. We also tested our tool on whole-slide images (WSI) with clinical annotations, investigating whether there is a correlation with clinical outcome, which has been shown for this patient cohort previously by manual counting [9, 10]. Furthermore, we also tested our approach on tissue microarrays (TMAs) to check whether this could be used for the assessment of tumor budding.

## Methods

### Patient specimens and raw data generation

#### Specimen and data management

Whole-slide tissue specimens of formalin-fixed paraffin-embedded tumor tissue (*n* = 20 whole tissue slides) and TMAs of tumor tissue were retrieved from the pathology archive of the Institute of Pathology (Medical University of Graz, Austria). These cases belong to a previously published patient cohort of 381 patients (166 males, 215 females; median age 70.1 years) used and described by Harbaum et al. [9]. All procedures were carried out in accordance with the Declaration of Helsinki and in accordance with the local ethics committee (decision 18–199 ex 06/07).

All cases have been included in the study in a completely anonymized way with unique identifier for case (e.g., “GraMa001”), sample (which corresponds to tissue type) (e.g., “Samp001”) and TMA core (e.g., “Core0001”). In the end, every measurement has one complete unique composite identifier, such as “GraMa001-Samp001-Core0001”. The clinical information includes age, gender, TNM stage, number of infiltrated lymph nodes, grading and recurrence time.

#### Staining and digitalization

The tissue blocks underwent routine histochemical (HE) and immunohistochemical staining for pan-cytokeratin (cytokeratin, clone AE1/AE3, M3515, Dako/Agilent, Santa Clara, CA, USA) [22, 23]. The resulting sequential sections were digitalized as whole-slide images (WSI) using a digital microscope and M8 scanner (PreciPoint GmbH, Freising) and saved after conversion as svs files on a local Omero server [24].

### Image processing in general

Image processing was performed in MATLAB (R2017a) on a desktop PC (Windows 7 Enterprise, Intel Core i7–4790, 32GB RAM, NVIDIA GeForce GT 630).

MATLAB-coding was carried out in accordance with style guidelines proposed by Johnson to increase the readability [25, 26]. Furthermore, object-oriented programming was applied [27], and speed up guidelines by Altman were followed [28].

In summary, all images underwent image modifying processing steps as part of the analysis, which are mentioned within the text and the legends in accordance with Digital Image Ethics [29].

### Convolutional neural network training and application in general

To decide whether a tile (a tumor bud proposal) contained a single tumor bud or not, we used MatConvNet by Vedaldi et al. as CNN-toolbox in MATLAB [30]. For this classification task, we constructed an 8-layer CNN (see Additional file 1: Table S1). It was trained on a data set of 6292 images (100 × 100 pixel). These data set had been manually labeled by a pathologist (CAW). The dataset is available on HeiData.

The training was performed for 10,000 epochs with a constant learning rate of 10–5 on the BwUniCluster (state of Baden-Württemberg, bwHPC).

### Tissue microarray (TMA) image processing

The MATLAB code of the method described below is available on GitHub (DOI: 10.5281/zenodo.1300211). It comprises tools tested for a scientific approach.

#### Data access and image registration

On the basis of the MATLAB Omero toolbox [24, 31], thumbnails from two WSIs (HE- and IHC-stained) were loaded into MATLAB’s workspace (Additional file 2: Figure S3).

By using color thresholding, the TMA cores were separated from the background, the images were converted to binary images and the objects were automatically counted and named per image (Additional file 3: Pseudocode 1).

Subsequently, the thumbnails were registered by the MATLAB built-in SURF-based registration. These registration results were visually checked and in the case of an obviously wrong registration, a manual control-point-based registration was applied. By doing so, the corresponding core pairs could be consolidated for their numbers and positions in both images (Additional file 3: Pseudocode 2).

#### Download of the TMA cores

On the basis of the consolidated core pairs, every single core could be loaded from the Omero server in full resolution and locally saved as TMA core object (containing the slide ID, the core ID, the core position and an HE and IHC image of the core).

#### Core analysis

The pan-cytokeratin-stained cores were analyzed by a custom-written MATLAB tool to detect tumor buds, defined as small, independent clusters of 1–5 poorly or undifferentiated tumor cells:

(i) A custom-written implementation of color deconvolution was applied to separate the background and foreground staining [32, 33].
(ii) The intensity information for the brown component was thresholded by k-means clustering for back- and foreground and thus converted into a binary image (Additional file 3: Pseudocode 3).
(iii) The detected objects in the binary image underwent two steps of clustering. First, objects with near coordinates and equal morphology (area, perimeter) were combined. Thereby, huge tumor areas with unequal staining were recombined. Second, objects were clustered in regard to their border distance and area. By doing so, small tumor fragments that were in close proximity to a huge tumor mass were included with that mass (Additional file 3: Pseudocode 4).
(iv) Then, objects were classified in regard to their size; if they were too small or too big to be a tumor bud, they were discarded. Next, they were classified by a custom-trained convolutional neural network (CNN; MatConvNet by Vedaldi et al. [30]) to discard objects that did not show the expected morphology. The (completely anonymous) training and validation data is available on heiDATA.
(v) Finally, for the resulting labeled images, the number, size, shape, etc. of the objects could be calculated by MATLAB built-in functions [34]. Furthermore, the spatial distribution and the distances of all objects to their neighbors were calculated on the basis of Delaunay triangulation [34]. In contrast to other works on automatic tumor bud detection, we relied only on pan-cytokeratin-positive area and size [12].

### Whole-slide image (WSI) image processing

#### Tumor and tumor border region of interest generation

On the pan-cytokeratin-stained WSI (*n* = 20) a region of interest (ROI) for the complete tumor and the border zone tumor-surrounding tissue was manually drawn in a local Omero client [24]. The corresponding ROI data were loaded by the Omero-MATLAB-toolbox [31] into the local MATLAB workspace.

#### Generation of virtual TMA (vTMA) cores

A grid with a grid point distance of half the mean TMA core diameter (1800 pixel) was drawn on the WSI (compare Fig. 3). Thereby, every grid point corresponded to the center of one virtual TMA (vTMA) core. Subsequently, all grid points within the above specified ROI were loaded on the basis of the Omero-MATLAB-toolbox and saved as a TMA core object (containing the slide ID, the core name and position, and an IHC image of the core).

#### vTMA core analysis

Due to different times of staining (TMA slides were stained in 2016, whole-slide cases were stained in 2017) there were staining differences between the two batches (initial TMA slides and whole tissue slides). To overcome this, Rheinard stain normalization was applied in a MATLAB implementation by Manohar P. Kuse [35–37]. However, best results were obtained with k-means clustering of the colors and CNN evaluation without color adaption.

Subsequently, the virtual cores underwent the same image processing workup as described above for the TMA cores (section “Convolutional neural network training and application in general”).

### Data management

As described above, image processing was performed in the MATLAB environment. The results were saved in Excel spreadsheets (Microsoft Excel 2010, Microsoft Corporation, Redmond, WA, USA).

The clinical information and the TMA slide information (link between TMA core position and patient ID) were also saved in Excel spreadsheets. For the latter, we manually combined the automatic MATLAB generated core numbers (referred to as MATLAB core IDs) with the real-world IDs on a schematic illustration of the TMAs in a (humorously Rosetta Stone-like) Excel spreadsheet.

The information was gathered in different R databases [38]: one database for the patient level and one database for the core level. Statistical analyses were performed in R version 3.2.4 [38].

Major parts of the image processing data are also freely available on heiDATA.

### Monte Carlo simulation

To determine what sample size, how many randomly distributed cores per case were needed to reassemble the cases’ characteristics, we ran a Monte Carlo-like analysis. Therefore, (i) from every case, a predefined number of vTMAs were randomly (10 times per case and sample size) selected and then the median number of buds, the median budding score and the normalized Shannon entropy were calculated [39–45]; (ii) then the number of random samples (*n* = [2:1:200]) was changed and step i was repeated.

By doing so, we obtained a range of expected values for every sample size or relative sample size (normalized to the total number of slides per case).

## Results

### Is there a correlation between the human estimation-based budding score and clinical parameters within the analyzed patient cohort?

**Fig. 1:**
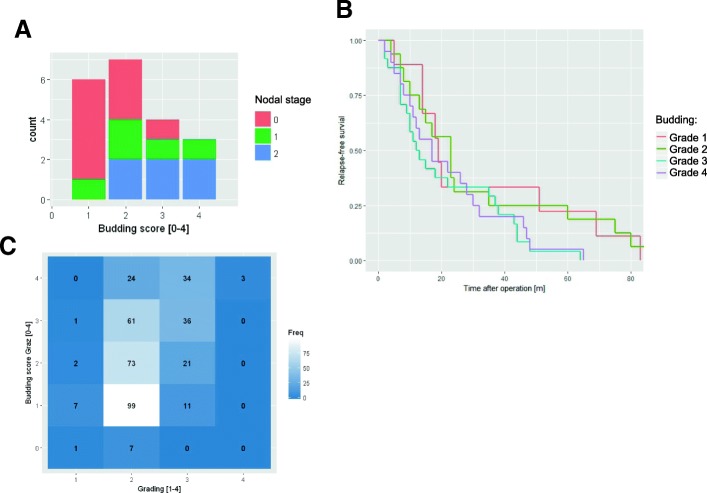
Correlation of the budding score to clinical data. The externally provided budding score of 328 cases showed a correlation to the patient’s nodal status (**a**) and to the regression-free survival (**b**), as previously published by [9, 10]. Furthermore, within the database there is also a significant correlation between the budding score (grades 1 to 4 with the intervals [0–5], [5–17], [17–20] and [20-∞] to stratify the absolute number of tumor buds [9]) and the morphology-based tumor grading (Grade 1–4) (**c**)

As previously published by Harbaum et al. [9] and Max et al. [10] on the herein analyzed patient database, there was a correlation between a high budding score based on visual estimation on whole-tissue slides to clinical parameters such as positive nodal status and inferior regression free survival. By using their data and sample set, we could independently confirm the previously published correlation between nodal status and budding score (Fig. 1a) (*p* < 0.05), as well as the correlation between the budding score and regression-free survival (Fig. 1b). As a new analytical feature, we could also find a correlation between the budding score and morphologic tumor grading within these datasets (Fig. 1c) [8].

### Can we define a reliable and reproducible automatic image processing approach?

There is no common generally valid definition of tumor budding in the literature. We decided to use the definition established by Satoh et al. [13], in which they defined tumor budding as “cancer cell nests of fewer than five cells in the interstitium” with subsequent grouping of budding in an interval of 5 grades (grade 0 with 0 buds, grade 1 with 1–5 buds, grade 2 with 6–10 buds, grade 3 with 10–19 buds and grade 4 with ≥20 buds). This work was chosen because of its linear grading intervals for stratification of tumor buds.

**Fig. 2:**
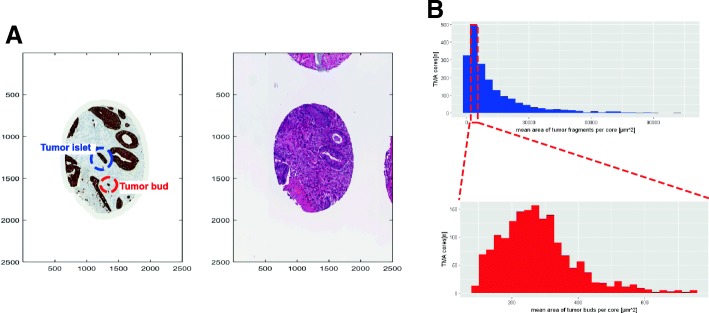
Definition and detection of tumor buds. **a** Example single TMA core stained for pan-cytokeratin and hematoxylin and eosin (HE). The red circle highlights a tumor bud next to larger tumor island. The blue circle is a small tumor part, which we referred to as a “tumor islet”; which is a small tumor aggregate but does not meet all criteria for a tumor bud. **b** Histogram of pan-cytokeratin positive tumor area per core (upper plot) and pan-cytokeratin tumor bud area per core (lower plot). One can see that the mean area of all tumor fragments (upper panel) shows a huge dispersion (x-axis 0–100,000), whereas the mean area of the tumor buds is less distributed (x-axis 0–1000)

Transferred to our image processing approach, this corresponds to a stained/brown area of 72–750 μm^2^ (300–3125 pixels) as a threshold for tumor buds (compare red circle in Fig. 2 A and histograms of the area of tumor objects in Fig. 2b). On the basis of this definition, potential tumor buds could be separated from other small tumor aggregates, which we referred to as tumor islets and which are larger in size. Since this area-based definition is prone to size variation (e.g., clusters of more than 5 very small tumor cells or stained area without nuclei) or staining variations (e.g., big structures are unequally stained leading to several stained spots in one structure), further validation steps were applied.

The detected tumor buds underwent further evaluation by cluster analysis (in regard to size, shape and border distance) and by a convolutional neural network (custom-trained MatConvNet [30]) to reduce the false positive rate. By doing so, small islets of positive staining within whole tumor mass were no longer recognized as tumor buds.

Our detection method (details in section “Tissue microarray (TMA) image processing”) was optimized and validated on 20 test cores (10 real TMA cores (rTMA) and 10 virtual TMA cores (see section “Is there a correlation between the number of tumor buds and the budding score to the nodal status in virtual TMA-cores?”)). We initially marked tumor buds manually on images of these cores (“ground truth”) and compared these findings to the automatic segmentation; regarding the absolute number of tumor buds per core, there were more discrepancies, especially for cores with high numbers of tumor buds, but still an R^2^-value of 0.86 was achieved. With one exception (budding score 3 instead of 2), we achieved an R^2^-value of 0.96 (perfect correlation) for manual vs. automatic evaluation.

### vTMA as a method to represent whole slide image analysis

#### Is there a correlation between the number of tumor buds and the budding score to the nodal status in virtual TMA-cores?

**Fig. 3:**
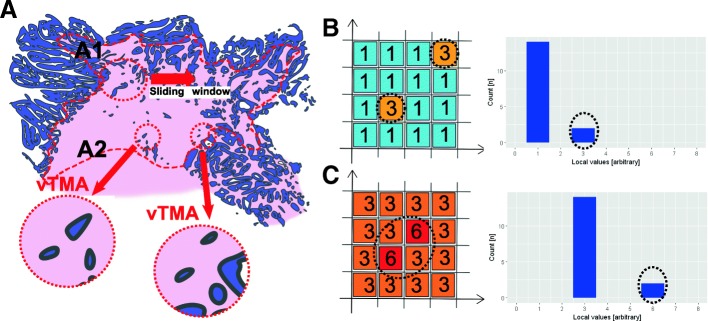
Sketch of the analysis. A) ROI tumor (not shown) and the ROI border (dashed red line) were manually delineated by a pathologist (CW). A1: **a** sliding window moved over the ROI border and cropped every half TMA diameter in the underlying, TMA core-sized image. A2: The resulting tile (exactly the size of one TMA core) was then defined as a virtual TMA (vTMA). **b**, **c** This procedure leads to a value (number of tumor buds or budding score) per vTMA. This spatial data could be plotted as a heat map (example heat maps with arbitrary values in the left part) or as a histogram (right part of the figure)

We randomly selected 20 cases from our cohort (pN0 (*n* = 9), pN1 (*n* = 5), pN2 (*n* = 6)) and digitalized pan cytokeratin-stained whole slides, with manually delineated tumor areas (“ROI tumor”) and tumor invasion fronts (“ROI tumor border”). These regions were used to create virtual TMA (vTMA), which were TMA core sized tiles cropped from the whole-slide image (Fig. 3 A and section “Tissue microarray (TMA) image processing”).

**Fig. 4:**
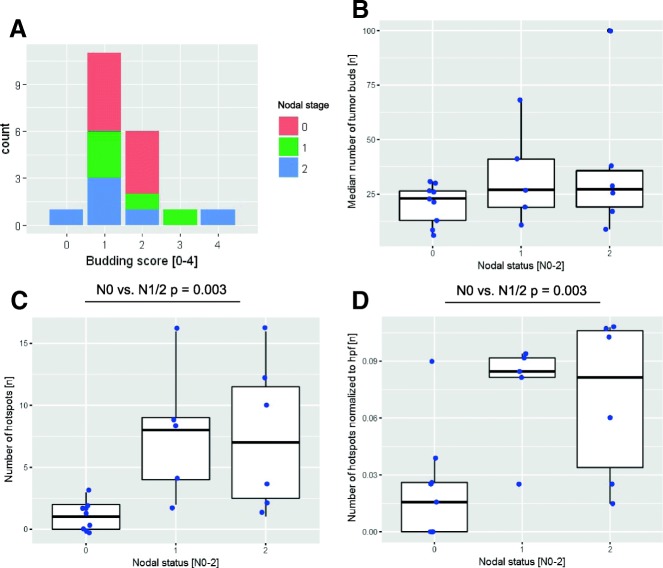
Analysis of vTMAs from 20 cases. From 20 selected cases (pN0 (*n* = 9), pN1 (*n* = 5), pN2 (*n* = 6)) one pan-cytokeratin-stained slide was digitalized and disassembled into virtual, overlapping vTMA cores (*n* = 290 ± 152). **a** No significant correlation was detected between the resulting budding score and the nodal status and **b** the median number of tumor buds within the 10 hottest spots and the nodal status for the complete ROI border. **c**, **d** Significant positive correlation between the nodal status and the absolute number of significant budding hotspots and the normalized number of significant budding hotspots. The latter is done to compensate for a trend toward higher tumor areas on the WSI for pN1 and pN2 cases

Comparing image processing results with human estimation-based data from previous publications [9, 10] for these 20 cases showed only a weak correlation for median budding (data not shown). Furthermore, there was no significant correlation for the median number of tumor buds in the ROI border or for the 10 hottest spots within the ROI border. The latter (10 hottest spots) was implemented according to Koelzer et al., who proposed to focus on the 10 hottest spots with the highest budding activity. There is also no correlation between our automatically obtained budding score and the median number of tumor buds (Fig. 4a). Furthermore, we found no correlation between the obtained budding score and the nodal status (Fig. 4b).

#### Is there a difference regarding hotspots for pN1/2 cases vs. pN0 by vTMA?

Dealing with spatial data, we plotted our measurements per vTMA in relation to their coordinates on the slide and interpolated between the measurement points. To carve out significant hotspots, we calculated the Z-score for every measurement with the formula (*x* − *μ*)/*σ*), where x is the local value, μ is the mean value and σ is the standard deviation. Then, we plotted the Z-score values against the coordinates (heat maps in Additional file 4: Figure S2) and finally defined hotspots as areas with a Z-score > 1.67 (in parallel to our previous work on angiogenic hotspots in CRC [33]).

By doing so, the number of significant budding hotspots (areas where the number of tumor buds is significantly different in regard to the overall distribution of that case) could be calculated per slide. The number of budding hotspots normalized to the analyzed area significantly correlated with the nodal status (Wilcoxon rank sum test *p*-value = 0.031) (Fig. 4c-d). Modeling the nodal infiltration (present or absent) by logistic regression led to good fit with an area under the curve (AUC) of 0.838.

#### Is there spatial heterogeneity for budding and nodal status in whole-slide images by vTMA?

The above described data indicates that the number of budding hotspots (calculated in comparison to the underlying distribution) and not the underlying values themselves show a correlation to the nodal status; for example, in the sketch in Fig. 3b, the heat map has a mean value of 1.25 and the two tiles with an arbitrary value of 3 are significant hotspots in relation to their background. The heat map in Fig. 3c has a mean value of 3.75 and the tiles with an arbitrary value of 6 are significant hotspots. Thus, the absolute value per tile does not define our approach a hotspot, but the relation of the tile value to the rest. In addition, these hotspots are defined by the distance of the tiles. For example, both heat maps in Fig. 3b-c had two significantly different tiles, but only in Fig. 3b are they spatially separated and therefore forming two hotspots and not one as in Fig. 3c. The histograms for both example heat maps have equal statistical distributions. In conclusion, it seems to be a problem of spatial heterogeneity.

Since the spatial information is lacking for the later rTMA analysis in sections, we checked whether features describing the heterogeneity, calculated on the basis of the histogram, were able to predict the nodal outcome. First, we separated the histogram on the basis of the Z-score into measurements within and outside the normal distribution of the cases (histograms in Additional file 4: Figure S2) and then analyzed the values outside the normal distribution; no correlation to the nodal status was found for the resulting number of significant vTMAs normalized to the overall number of vTMAs per slide (*n* = 0.07 ± 0.02, *n* = 0.07 ± 0.01 and *n* = 0.07 ± 0.01), the median number of tumor buds per vTMA (*n* = 19.50 ± 7.46, *n* = 26.40 ± 17.60 and *n* = 31.67 ± 31.50), or the maximum number of tumor buds per vTMA (*n* = 26.44 ± 10.57, *n* = 45.20 ± 33.32 and *n* = 46.50 ± 36.74). The latter, interestingly, is in contrast to the work by Koelzer et al. [11] proposing to focus on hotspots. Similarly, comparing the median and maximum budding score for these vTMAs outside the normal distribution showed no correlation.

**Fig. 5:**
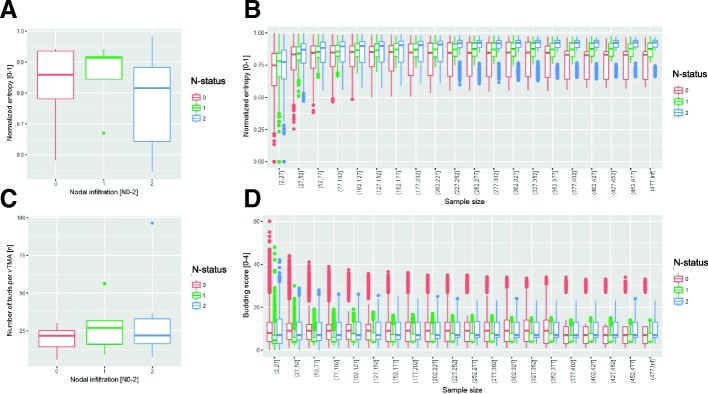
Monte Carlo-like simulation. From every case (of the 20 cases with 290 ± 152 vTMAs from the tumor border) repetitively a predefined number of vTMAs were chosen at random, and subsequently the median number of buds, the median budding score and the normalized entropy were calculated. This process was repeated several times (*n* = 10) with different sample sizes (from *n* = 2 to *n* = 200). **a** and **c** show the normalized entropy and the median number of tumor buds in relation to the sample size, respectively. **b** and **d** show the normalized entropy and the median number of tumor buds for the complete tumor border, respectively

Second, we calculated the histological Shannon’s entropy [40, 41] as proposed by Kayser et al. [42–45] and as previously applied by us to describe the spatial heterogeneity in thymus specimens [39]. The entropy normalized to the sample size (Fig. 5a and c) showed a no significant trend towards higher entropy for pN1 and lower entropy for pN2 (pN0 0.83 ± 0.13 bit, pN1 0.86 ± 0.11 bit and pN2 0.78 ± 0.17 bit).

#### Summary of vTMA analysis

In summary, within the subcohort of 20 cases, there were no significant correlations among the absolute number of tumor buds, the budding score and their statistical derivates (e.g., median values). Accordingly, by means of our established tumor bud detection, we could not reproduce for whole-slide images the previously published results that were based on human evaluation [9, 10].

However, we could show a significant correlation between the number of significant budding hotspots and the nodal status for that subcohort. Since a hotspot definition based on histogram analysis only failed to show a significant correlation, we defined hotspots on the basis of spatial statistics by taking the relation of a local value to the remaining analyzed field (histogram analysis) and by taking the position of measurement values (spatial information) into account.

### Analysis of real TMA (rTMA)

#### What is a reasonable number of TMA cores per case to reproduce whole-slide analyses?

Regarding rTMA data, we tested how many TMA cores per slide were needed to reassemble the cases’ characteristics, especially in regard to their heterogeneity. Therefore, we ran a Monte Carlo-like simulation on the basis of our vTMA data from the tumor border (compare section “rTMA: Correlation between ground truth and TMA-based budding score”).

As expected, the simulation showed that the results for normalized entropy (Fig. 5b) and for the median number of buds per core (Fig. 5d) align to the overall results (Fig. 5a and c) with increasing relative sample size.

#### rTMA: Correlation between ground truth and TMA-based budding score

As mentioned above, in previous works, the budding score was evaluated by a pathologist for this patient cohort [9, 10]. Therefore, we defined this budding score as ground truth for our work.

**Fig. 6:**
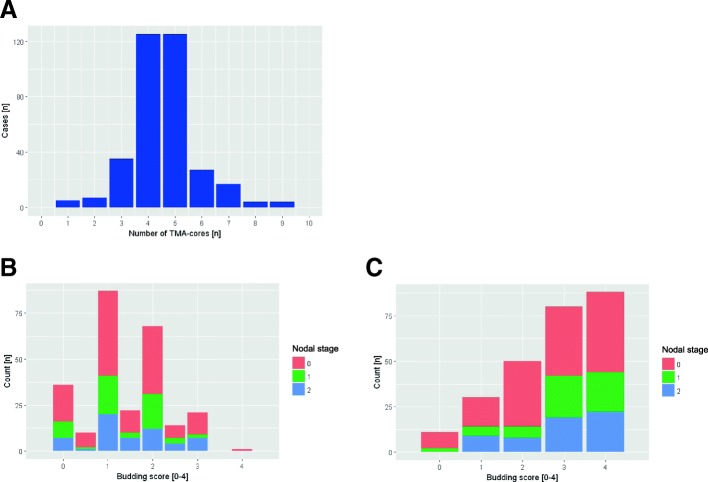
Correlation of the TMA data to clinical data. **a** Histogram of the number of TMA cores from the tumor region per case (*n* = 4 ± 2). **b**, **c** Histogram of the **b** median budding score per case [0–4] and the **c** maximal budding score per case. The nodal status [pN0–2] is added by color coding

In our rTMA data, the number of cores from the tumor region with *n* = 4 ± 2 was rather constant (Fig. 6a). However, in regard to the results of the Monte Carlo simulation, where the values began to close on the overall values for sample sizes > 100, these numbers are far too small to be representative.

Consequently, comparing to this human estimation-based ground truth, the median (accuracy = 0.208) and the maximum rTMA-based budding score (accuracy = 0.239) showed only a weak correlation. Furthermore, stratifying the median (Fig. 6b) or maximum (Fig. 6c) rTMA-based budding score to clinical data such as nodal status did not reveal any correlation.

Against this background, with more or less randomly sampled TMA cores without spatial information, calculating the number of significant budding hotspots (as previously described in section “Is there a difference regarding hotspots for pN1/2 cases vs. pN0 by vTMA?”) is not possible. Calculating the entropy on the basis of the number of cores per case and the obtained probabilities does not show significant differences in regard to the nodal status (pN0 0.57 ± 0.20 bit, pN1 0.60 ± 0.22 bit and pN2 0.57 ± 0.24 bit). In this context, changing the number of intervals and the cut-off criteria of the budding score also did not lead to a better distinction. The above-described algorithms were tested on a set of new tiles (*n* = 16) from different tissue blocks, staining and processing rounds after manual annotation in Fiji. These images had not been used in the process of CNN-training or method development. To avoid overlap and edge issues, these images contained only complete cells and the edge zones were blackened (compare black area in Fig. 3a).

For these 16 tiles, the results of the manual segmentation (as ground truth) were compared to the results of the automatic, CNN cascade-based detection on the basis of calculating the bounding box overlap (as described above). By doing so, the correct positive rate (0.87 ± 0.03), false positive rate (0.11 ± 0.04), false negative rate (0.11 ± 0.04), double detection rate (0.04 ± 0.01) and precision (0.88 ± 0.03) were calculated (compare Fig. 3c for tile #5).

## Discussion

### Comparison of the tumor budding definition and assessment in the literature with our approach

In literature, tumor budding is most often defined as (i) clusters of 1–5 poorly or dedifferentiated tumor cells at the invasive front of tumors, next to larger, circumscribed tumor formations [14, 15]; (ii) and needs to be strictly discriminated from a diffuse infiltrative growth pattern [1, 8, 16]. Our image processing approach is consistent with the abovementioned definition. It takes the size and the localization in relation to the main tumor masses into account and considers the tumor bud morphology through a convolutional neural network. Furthermore, it covers the problematic area of diffuse growth or infiltration pattern by the hierarchical clustering step, which sums up such formations as one object due to the shape and localization of its subparts (step iii in section “Convolutional neural network training and application in general”.3). Several works from different groups address tumor budding for CRC and its related role as a prognostic factor, in particular in regard to nodal status [8, 10, 16, 46–48]. Thereby, a plethora of different tumor budding assessments have been applied. For example, some groups have counted tumor buds in absolute numbers (e.g., Ueno et al. [48], Prall et al. [16]), others have stratified the absolute number into different grades (e.g., Max et al. [10]), and still others just defined high- and low-grade budding activity on the basis of the absolute numbers (Hase et al. [8]). Our approach primarily counted the number of tumor buds per high power field (one high-power field had approximately the size of a single TMA core). These numbers were then stratified into five budding grades (from grade 0 with no budding to grade 4 with extensive budding) in reference to previous works by Koelzer et al. and Satoh et al. [11, 13]. Of note, the findings of Koelzer and Satoh were mainly established based on HE staining, and their transferability to more sensitive IHC-based estimations is problematic. The higher sensitivity of the latter method could lead to higher grades [49].

In comparison to the results of a manually defined ground truth (blinded annotation by a trained pathologist, CAW), the automatic evaluation showed a very good accordance for the budding score and a good accordance for the overall number of tumor buds for whole-slide analysis (section “Tissue microarray (TMA) image processing” and Additional file 5: Figure S1), which opens the possibility of analyzing entire sections in a reliable and reproducible fashion.

### One spatial statistics derived definition of clinically significant budding hotspots

For a subcohort of 20 cases, we focused on the manually delineated infiltrative border in accordance with Caie et al., who also focused on infiltrative border and showed a correlation of immunofluorescence-based image processing-based tumor budding assessment with manual budding analyses and clinical parameters [12]. Surprisingly, for our data, we could not show a correlation between the median or maximum number of tumor buds and the nodal status. Additionally, we found no correlation between budding score and nodal status. Even focusing budding analyses on hotspots (as proposed by many researchers) did not lead to a significant stratification of cases in regard to the nodal status.

By applying methods of spatial statistics [50–53] to describe the spatial heterogeneity (as recently published for vessels in CRC [33], for lymphatic hyperplasia in the thymus [54] or for lymphatic infiltrates in the bone marrow [55]), we found significant accumulations of tumor budding foci independent of the overall frequency of tumor budding, which we called budding hotspots. The number of these budding hotspots and not their budding metrics (e.g., median budding score in the hotspots) did correlate with the nodal status. This leads obviously to the conclusion that TMAs are not suitable for analyzing tumor budding.

### Pros and cons of our automatic image processing and the ground truth

In addition to the nonnegligible hassle of counting tumor buds in a section, the reproducibility of human-based results and/or the training efforts to ensure such reproducibility are major limitations and hamper routine estimation of budding. Studies showed inter-observer variations in the range of kappa = 0.61–0.83 [56, 57]. However, a different human-based evaluation method using 10 high power fields (hpf) in the region with the highest density of peri-tumoral budding showed a slightly better reproducibility (kappa-values in the range 0.5–0.87) [11, 58].

Our image processing approach has been validated in terms of absolute budding number and budding score with good to very good accordance compared to manually drawn “ground truth” (Additional file 5: Figure S1). This offers the option for reproducible, time-saving whole-slide analysis and the resulting possibility of applying spatial statistics.

Interestingly, mimicking the strategies of the human evaluation (considering only the hottest spot or the 10 hottest spots) with our automatized method did not lead to significant results. Only the number of hotspots defined by spatial statistics correlated with nodal status.

## Conclusions

Tumor budding in CRC is a complex phenomenon for which the visual assessment by a surgical pathologist cannot be easily reproduced by automatic image processing. On the basis of a combination of image processing and machine learning, we found that not the absolute number of tumor formations classified as “tumor buds” within the infiltrative region but rather their spatial arrangement in significant hotspots and especially the number of such hotspots is clinically meaningful. Consequently, the advice for the surgical pathologist is to focus more on the spatial distribution (as kind of pattern diagnosis), rather than on the absolute number, of tumor buds.

## Additional files

Additional file 1: **Table S1.** Architecture of the applied CNN. The 8-layer CNN has been designed to classify (100x100x3 pixel) images to the classes “tumor bud” and “no tumor” bud. It consists of two block of a combination of convolutional, rectifier and pooling layers and a fully connected layer. (DOCX 14 kb)

Additional file 2: **Figure S3.** Finding the corresponding core on two separate TMA-slides. Thumbnail of an HE-stained (A) and a pan-cytokeratin-stained (B) TMA-slide. The green circle highlights the same core on both slides, which has due morphological variations different numbers by the image processing based automatic counting. (PDF 6510 kb)

Additional file 3: Pseudocode 1 create TMA-map. Pseudocode 2 combine TMA-maps of different staining. Pseudocode 3 image analysis part I. Pseudocode 4 image analysis part II. (DOCX 16 kb)

Additional file 4: **Figure S2.** Dealing with spatial heterogeneity by different means. Histogram: On basis of the Z-score the vTMAs with values outside the underlying normal distribution could be identified. By doing so the histogram for the number of tumor buds per vTMA could be binarized into vTMA within and outside. Overlay WIS and heatmap for the ROI border: Furthermore by plotting the Z-score values against the coordinates on the WSI, a heatmap with the hotspot-probability could be obtained. In this map values > 1.67 are regarded as significant. (PDF 4012 kb)

Additional file 5: **Figure S1.** Validation of the detection method on 20 test cores. In 10 real TMA cores (rTMA) and 10 virtual TMA-cores (vTMA) every tumor bud has been manually segmented in Fiji [23] as ground truth. (PDF 9248 kb)

## Abbreviations

CNN: Convolutional neural network
CRC: Colorectal carcinoma
rTMA: real TMA
TMA: Tissue micro array
vTMA: virtual TMA
