# Supplementary material for: Automatic evaluation of tumor budding in immunohistochemically stained colorectal carcinomas and correlation to clinical outcome
Source: Diagn Pathol. 2018 Aug 28;13:64. doi: 10.1186/s13000-018-0739-3 (PMC6114534; doi:10.1186/s13000-018-0739-3)
Supplement: Supplementary file 1 — Table S1. Architecture of the applied CNN. The 8-layer CNN has been designed to classify (100x100x3 pixel) images to the classes “tumor bud” and “no tumor” bud. It consists of two block of a combination of convolutional, rectifier and pooling layers and a fully connected layer. (DOCX 14 kb) [file 13000_2018_739_MOESM1_ESM.docx]

Table S1 Architecture of the applied CNN

The 8-layer CNN has been designed to classify (100x100x3 pixel) images to the classes “tumor bud” and “no tumor” bud. It consists of two block of a combination of convolutional, rectifier and pooling layers and a fully connected layer.

| **Layer** | **Type** | **Filter size** |
| --- | --- | --- |
| 1 | convolutional | 21x21x3x50 |
| 2 | rectified linear unit |  |
| 3 | pooling layer |  |
| 4 | convolutional | 3x3x50x50 |
| 5 | rectified linear unit |  |
| 6 | pooling layer |  |
| 7 | fully connected | 9x9x50x2 |
| 8 | Sigmoid layer / output |  |
