# Supplementary figures and images for: Automatic evaluation of tumor budding in immunohistochemically stained colorectal carcinomas and correlation to clinical outcome
Source: Diagn Pathol. 2018 Aug 28;13:64. doi: 10.1186/s13000-018-0739-3 (PMC6114534; doi:10.1186/s13000-018-0739-3)

# Supp. Figure 3

## TMA HE

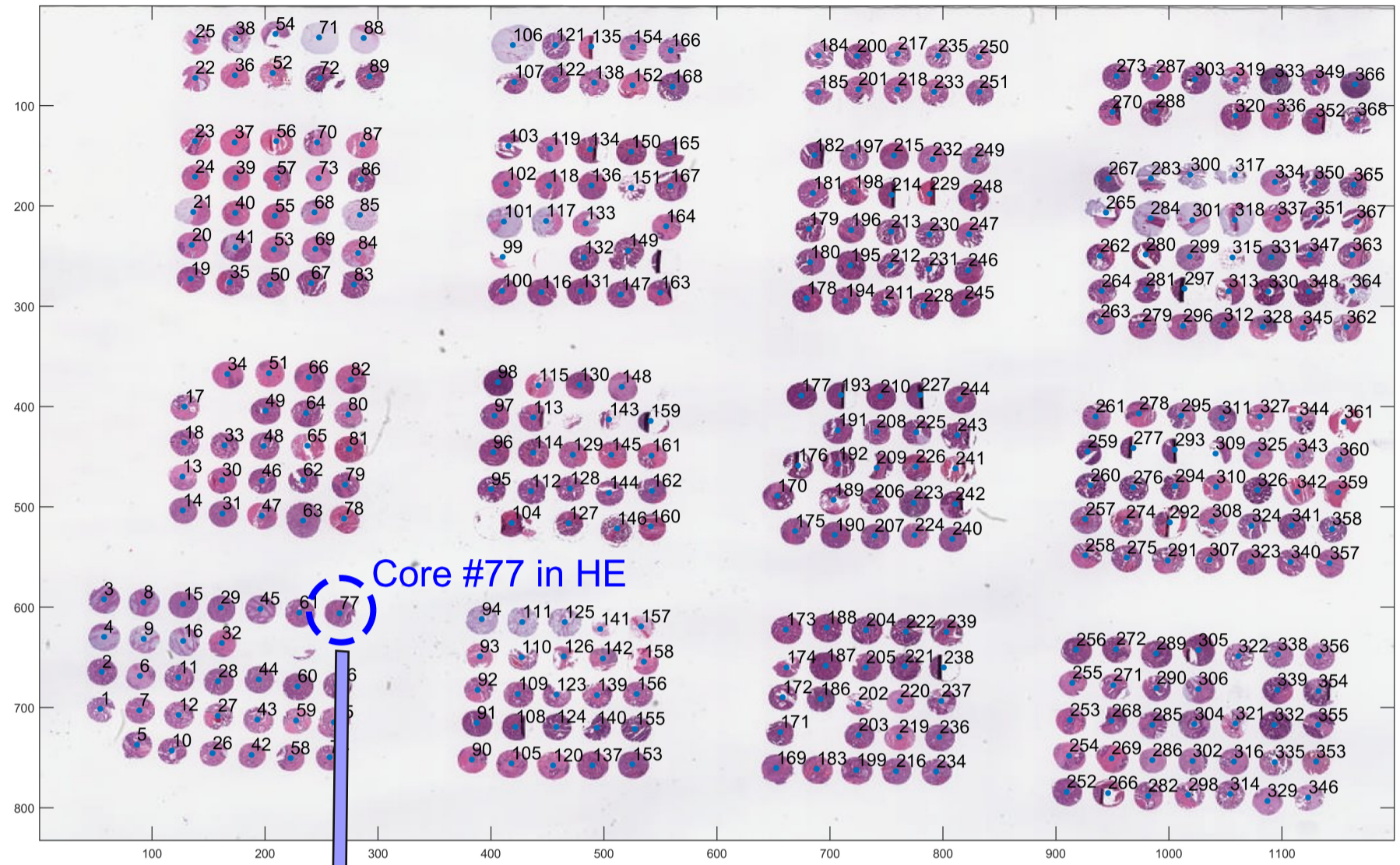

## TMA IHC

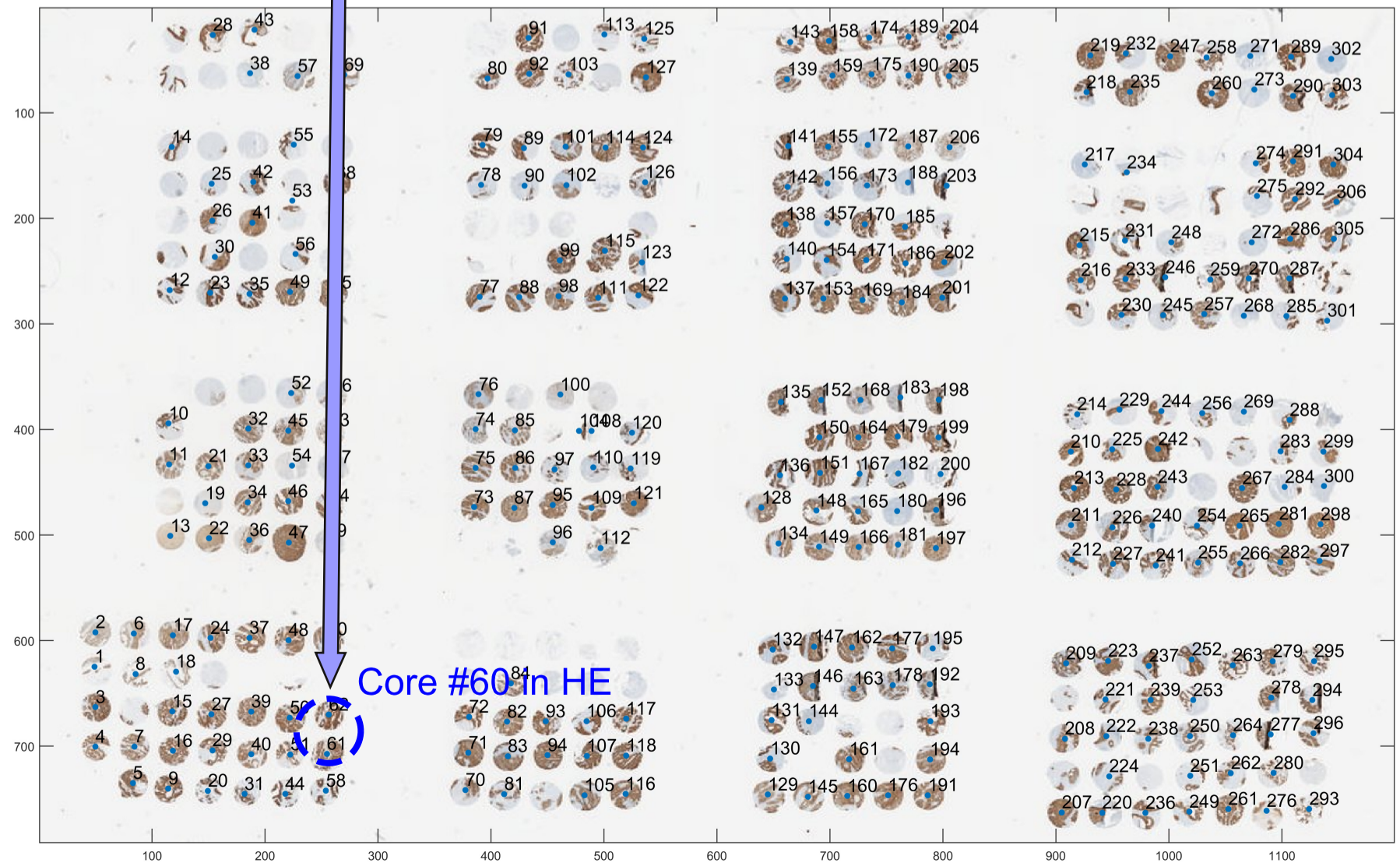

Supplement: Supplementary file 2 — Figure S3. Finding the corresponding core on two separate TMA-slides. Thumbnail of an HE-stained (A) and a pan-cytokeratin-stained (B) TMA-slide. The green circle highlights the same core on both slides, which has due morphological variations different numbers by the image processing based automatic counting. (PDF 6510 kb) [file 13000_2018_739_MOESM2_ESM.pdf]

Supp. Figure 2

Nodal infiltration stage N0

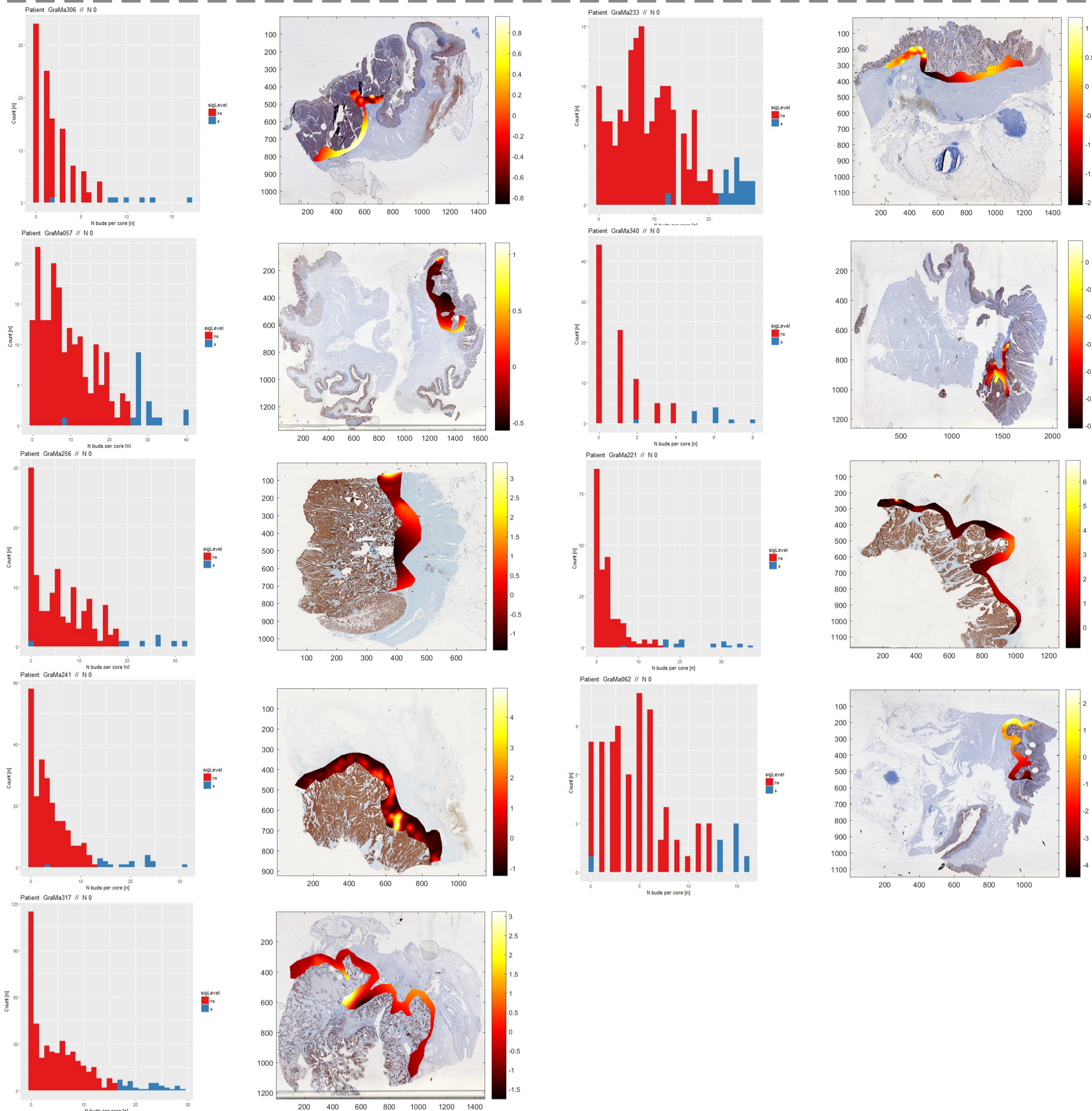

Nodal infiltration stage N1

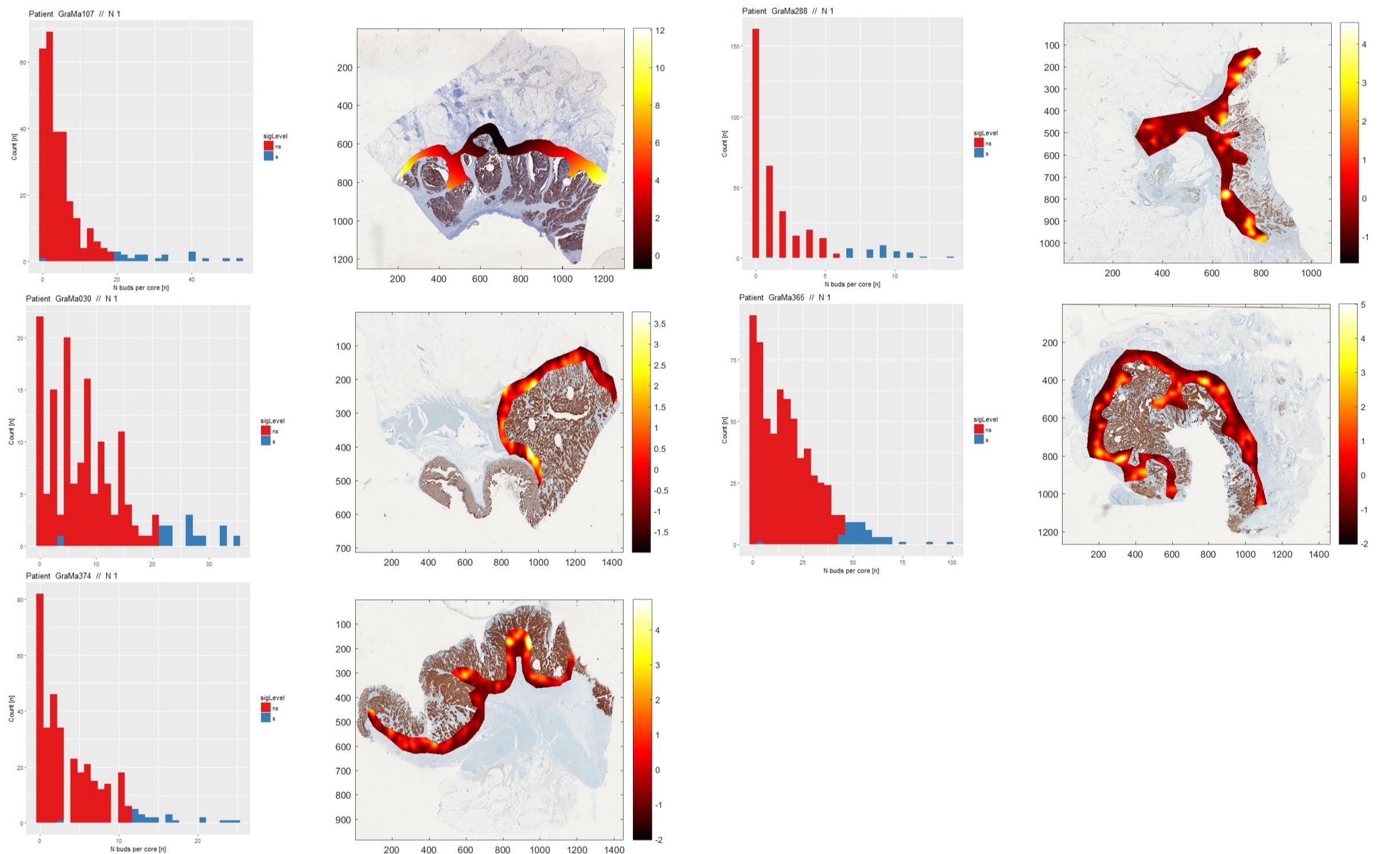

Nodal infiltration stage N2

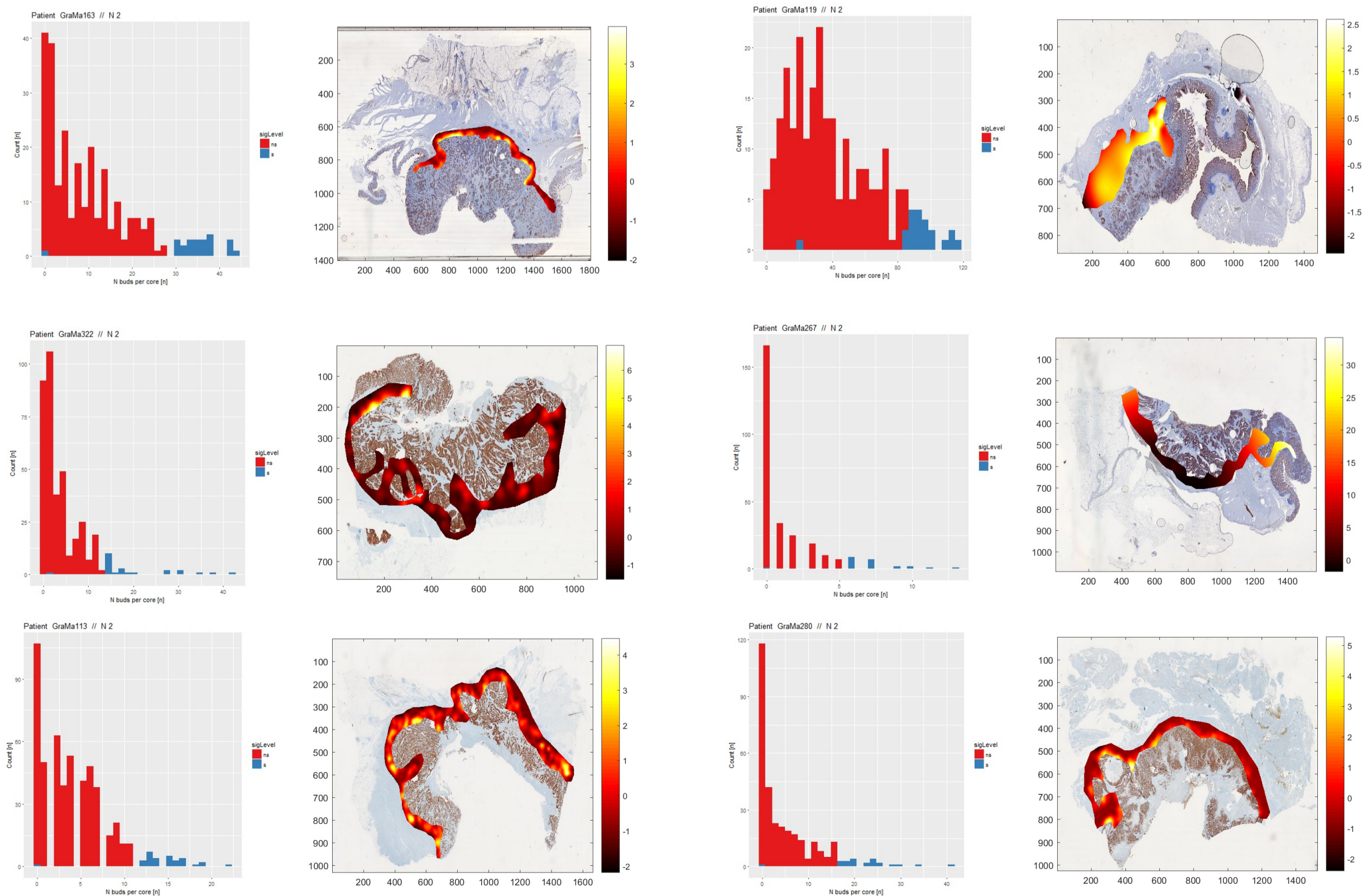

Supplement: Supplementary file 4 — Figure S2. Dealing with spatial heterogeneity by different means. Histogram: On basis of the Z-score the vTMAs with values outside the underlying normal distribution could be identified. By doing so the histogram for the number of tumor buds per vTMA could be binarized into vTMA within and outside. Overlay WIS and heatmap for the ROI border: Furthermore by plotting the Z-score values against the coordinates on the WSI, a heatmap with the hotspot-probability could be obtained. In this map values > 1.67 are regarded as significant. (PDF 4012 kb) [file 13000_2018_739_MOESM4_ESM.pdf]

Supp. Figure 1

TMA cores

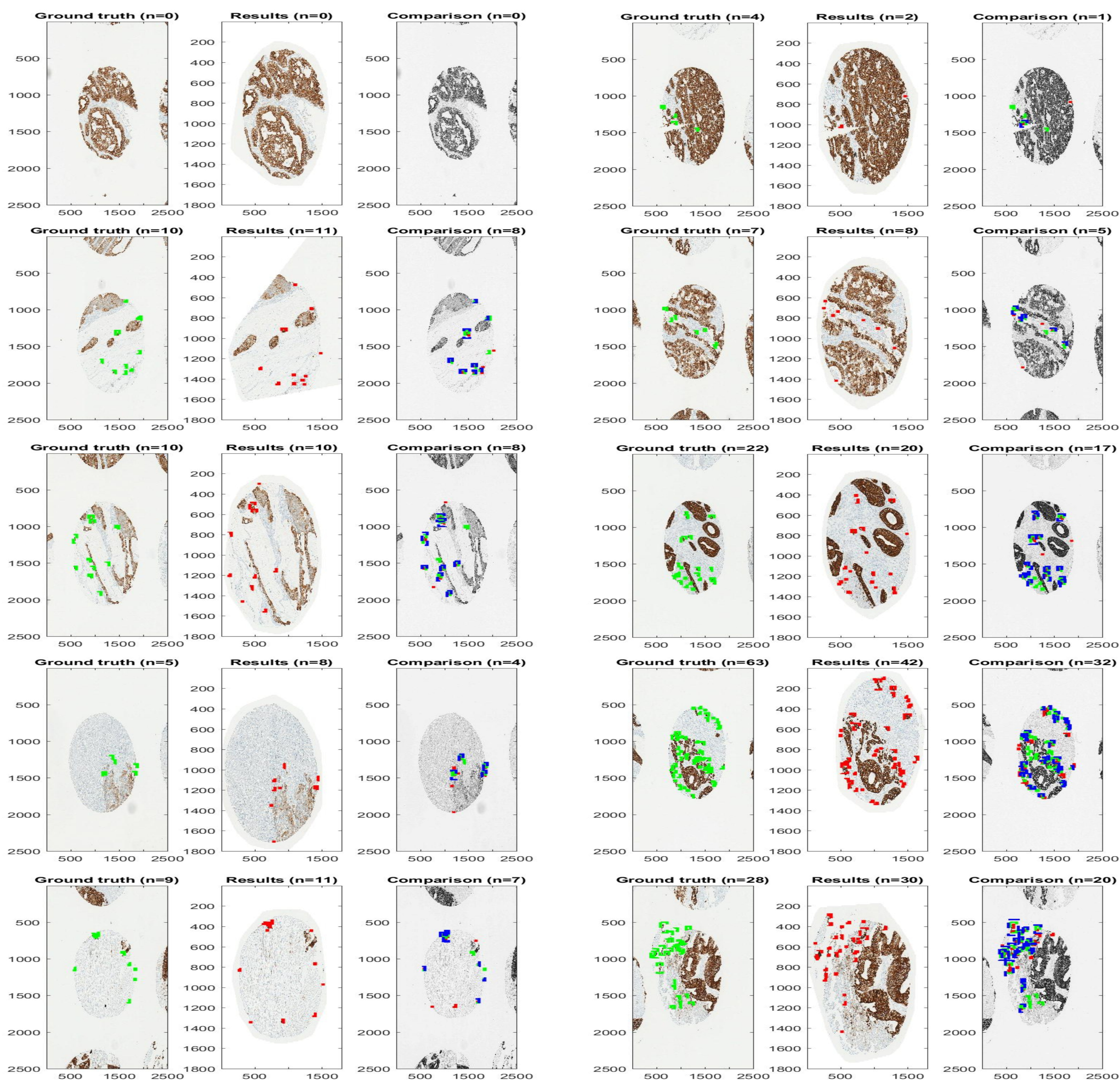

VTMA cores

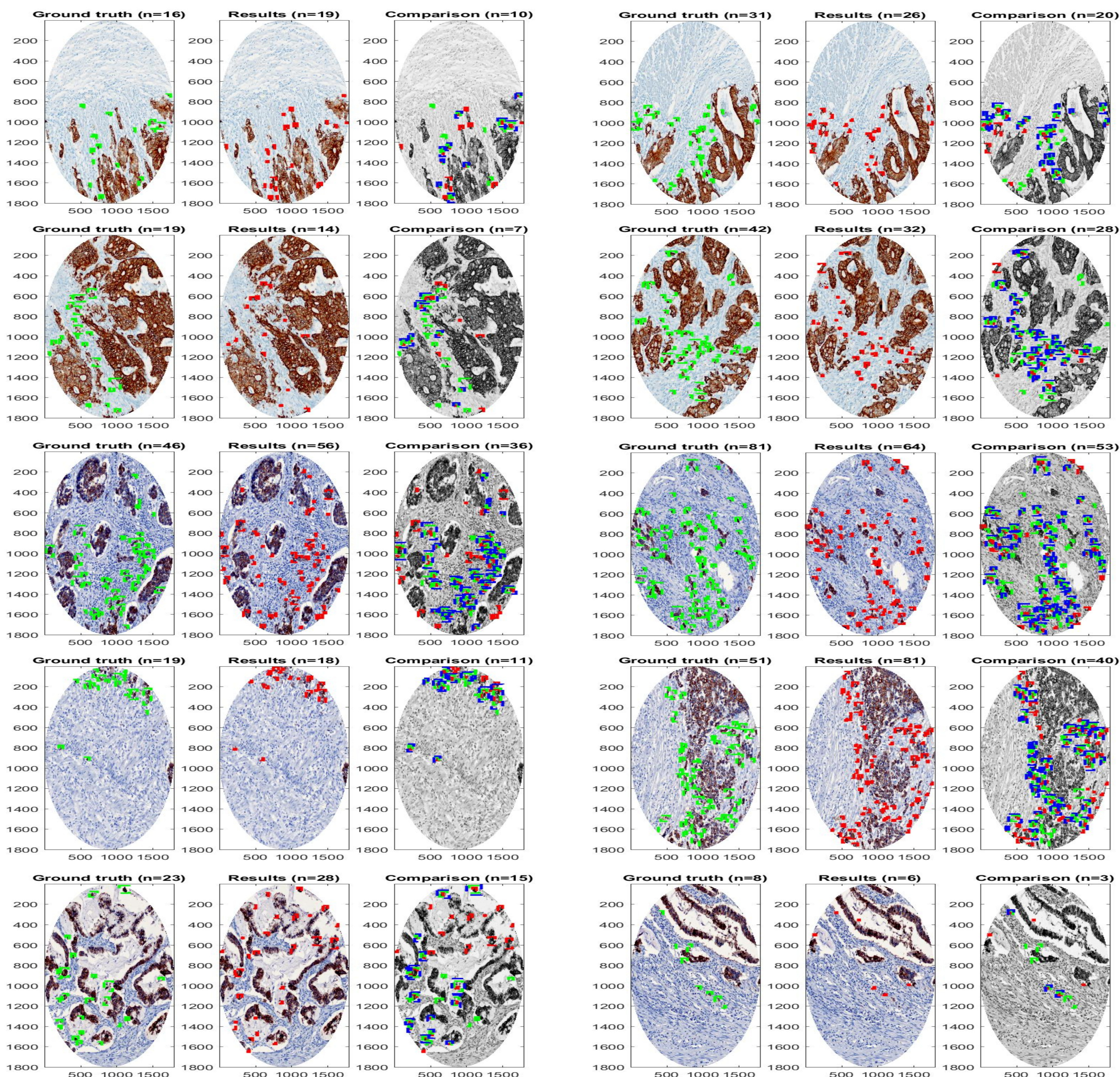

Supplement: Supplementary file 5 — Figure S1. Validation of the detection method on 20 test cores. In 10 real TMA cores (rTMA) and 10 virtual TMA-cores (vTMA) every tumor bud has been manually segmented in Fiji [23] as ground truth. (PDF 9248 kb) [file 13000_2018_739_MOESM5_ESM.pdf]
