# Supplementary material for: Automatic evaluation of tumor budding in immunohistochemically stained colorectal carcinomas and correlation to clinical outcome
Source: Diagn Pathol. 2018 Aug 28;13:64. doi: 10.1186/s13000-018-0739-3 (PMC6114534; doi:10.1186/s13000-018-0739-3)
Supplement: Supplementary file 3 — Pseudocode 1 create TMA-map. Pseudocode 2 combine TMA-maps of different staining. Pseudocode 3 image analysis part I. Pseudocode 4 image analysis part II. (DOCX 16 kb) [file 13000_2018_739_MOESM3_ESM.docx]

# SUPPLEMENTAL

Pseudocode 1: create TMA-map

input variable: omero-slideID

output variable: thumbnail of the TMA-slide

unique coreID per slide

coordinates of every core

thumbnail = download the thumbnail of the WSI

binary map = perform color deconvolution and thresholding

coordinates and core ID = apply MATLAB-built in black and white functions

**Pseudocode 2: combine TMA-maps of different staining**

input variable: TMA-maps of two sections from one specimen

output variable: matched unique coreID for both slides

matched coordinates for both slides

thumbnails = download the thumbnail of the WSI of different staining

coordinates and core ID = apply MATLAB-built SURF-based registration on the thumbnails

if registration failed (due to visual inspection)

perform manual control-point-based registration

matched coordinates = apply spatial transform on coordinates and cluster nearby points

to one measurement

**Pseudocode 3: image analysis part I**

input variable: picture of one pan-cytokeratin-stained TMA-core

output variable: imageBW

imagePrepared= pre-processing of the image (2D-median filtering)

imageForeground = color deconvolution to split up into a brown(foreground)-channel and
a blue(background)-channel

imageBW = thresholding of imageForeground and morphological image processing to reduce noise and combine fragmented objects

**Pseudocode 4: image analysis part II**

input variable: imageBW

output variable: number of objects

object size, perimeter, distance to neighbors

expectedObjectSize= 300-3125pixels (72-750µm^2^)

if objectSize ~= expectedObjectSize

discard objects

if CNN shows low probability for a tumor bud

discard objects

count and measure objects

perform Delaunay triangulation the get the centroid distances
